# Myocarditis With Ventricular Tachycardia Following Bivalent COVID-19 mRNA Vaccination

**DOI:** 10.1016/j.cjco.2023.05.006

**Published:** 2023-05-18

**Authors:** Jumpei Yamamoto, Toru Awaya, Takashi Nakagawa, Asuka Tamura, Yukio Hiroi

**Affiliations:** a Department of Cardiology, National Center for Global Health and Medicine, Tokyo, Japan; b Division of Cardiovascular Medicine, Toho University Ohashi Medical Center, Tokyo, Japan

**This report describes a case of probable myocarditis following bivalent COVID-19 vaccination. Although this was the patient’s fifth COVID-19 vaccination, it was the first dose of bivalent vaccine. The myocarditis may have been due to direct damage caused by an increase in free spike protein. This case demonstrates that postvaccination hypercytokinemia may cause myocarditis, leading to potentially fatal arrhythmia. Colchicine, which suppresses hypercytokinemia, may be effective in such cases and preferable to steroids, particularly in elderly patients. This adverse reaction is difficult to diagnose using conventional methods, and multimodality diagnostics are important, including myocardial scintigraphy and cardiac magnetic resonance imaging.**

## Case Presentation

The patient was an 81-year-old man who had been first admitted to our hospital for heart failure 9 years earlier due to diffuse left ventricular dysfunction (ejection fraction 30%). Coronary angiography revealed severe stenosis in the left circumflex branch #11, which was treated by percutaneous coronary intervention. Subsequent fatty acid analog iodine-123 beta-methyliodophenyl-pentadecanoic acid (^123^I-BMIPP). and thallium-201 resting myocardial scintigraphy showed no evidence of ischemia or infarction. Hypertensive heart disease was suspected due to a long history of hypertension. Holter monitoring showed a maximum of 12 beats of premature ventricular contractions from multiple origins but no sustained ventricular tachycardia (VT). In view of the patient’s wishes and financial considerations, an implantable cardioverter defibrillator (ICD) for primary prevention was not placed. A year and a half earlier, 1 month after his first COVID-19 vaccination, he had been readmitted to the hospital for heart failure and underwent coronary angiography, which showed no significant stenosis. Two weeks before his fifth COVID-19 vaccination, no worsening of his heart failure was detected at our regular outpatient clinic. However, on the day following bivalent BNT162b2 (wild and BA.4-5) vaccination (Pfizer–BioNTech, Cambridge, MA), he was rushed to our hospital with dyspnea.

**Figure 1:**
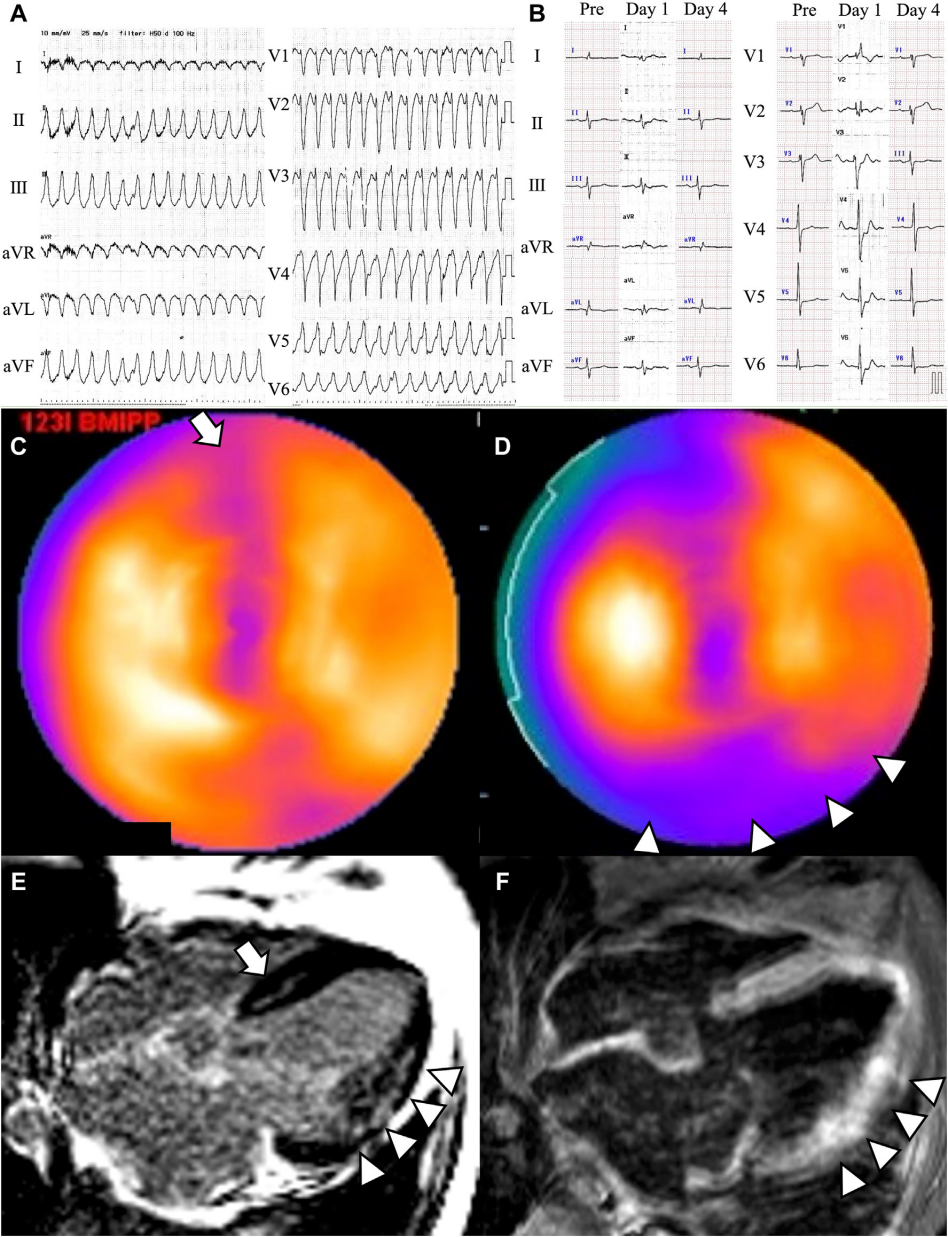
(**A**) Electrocardiogram obtained on arrival at our hospital shows a ventricular tachycardia waveform with a heart rate of 207 beats/minute. (**B**) Electrocardiogram recorded on day 1 shows new-onset right bundle branch block and ST-segment depression in leads V4–V6, which improved on day 4 to the prevaccination level. (**C**) Nine years earlier, a decrease in uptake of the fatty acid analog iodine-123 beta-methyliodophenyl-pentadecanoic acid (^123^I-BMIPP) had been noted in the anterior basal region (**arrow**), consistent with findings on cardiac magnetic resonance imaging (**arrow**). (**D**) On day 17, uptake of ^99m^technetium decreased from the basal to the mid-inferolateral region (**arrowheads**) under adenosine stress, consistent with the findings on cardiac magnetic resonance imaging (**arrowheads**). This new finding was not noted in the ^123^I-BMIPP scans performed 9 years earlier. (**E**) A cardiac magnetic resonance image (4-chamber view) demonstrating late gadolinium enhancement (LGE) of the inferolateral epicardial to mid layers and the anteroseptal mid layer (**arrow**), which indicates nonischemic myocardial injury. (**F**) A T2-weighted image showing high-signal areas of LGE in the lateral wall (**arrowheads**), indicating fibrosis and edema suggestive of myocarditis. The LGE sites in the anterior septum (**arrow**) do not show high signal in the T2-weighted image, suggesting preexisting cardiomyopathy.

When he entered the emergency room, he had cold extremities, a heart rate of 207 beats per minute, a systolic blood pressure of 74 mm Hg, and percutaneous oxygen saturation of 94% on room air. Arterial blood gas analysis showed a pH of 6.99, partial pressure of oxygen (PaO_2_) of 56.6 mm Hg, partial pressure of carbon dioxide (PaCO_2_) of 37.3 mm Hg, bicarbonate (HCO_3_^-^) 13.8 mEq/L, and lactate 12.2 mmol/L, indicating metabolic acidosis suggestive of cardiogenic shock. An electrocardiogram revealed left bundle branch block and right-axis deviation with sustained VT originating from the right ventricular outflow tract (Fig. 1A); this morphology of VT had not been seen on previous Holter monitoring. Pulseless electrical activity was seen after one cycle of synchronized electrical cardioversion, with return of spontaneous circulation after one cycle of cardiopulmonary resuscitation, including adrenaline administration and tracheal intubation with chest compressions by the emergency physician. He was admitted to the cardiology department and placed in the intensive care unit for systemic management. At this time, the electrocardiogram revealed new-onset right bundle branch block and ST-segment depression in leads V4–6 (Fig. 1B). Echocardiography revealed no change in left ventricular dysfunction and no thinning of the basal septum. A chest radiograph and computed tomography (CT) scans showed bilateral pleural effusions but no evidence of hilar lymphadenopathy, infection, or trauma on either side. Laboratory tests showed elevated levels of high-sensitivity troponin I (0.029 ng/mL; normal < 0.026 ng/mL) and new-onset liver dysfunction (aspartate transaminase 189 U/L, alanine transaminase 102 U/L). C-reactive protein (0.22-1.51 mg/dL) and brain natriuretic peptide (292.8-1066.7 pg/mL) levels were also elevated, in comparison with the outpatient laboratory test values. Cultures, COVID-19 polymerase chain reaction tests, and various viral antibody titers were negative. Collagen-related antibody titers, tumour markers, the free light chain κ/λ ratio, alpha-galactosidase A and angiotensin-converting enzyme activity, and the soluble interleukin (IL)-2 receptor level were normal. The VT was managed with amiodarone and his regular dose of β-blocker (carvedilol 7.5 mg/d).

**Figure 2:**
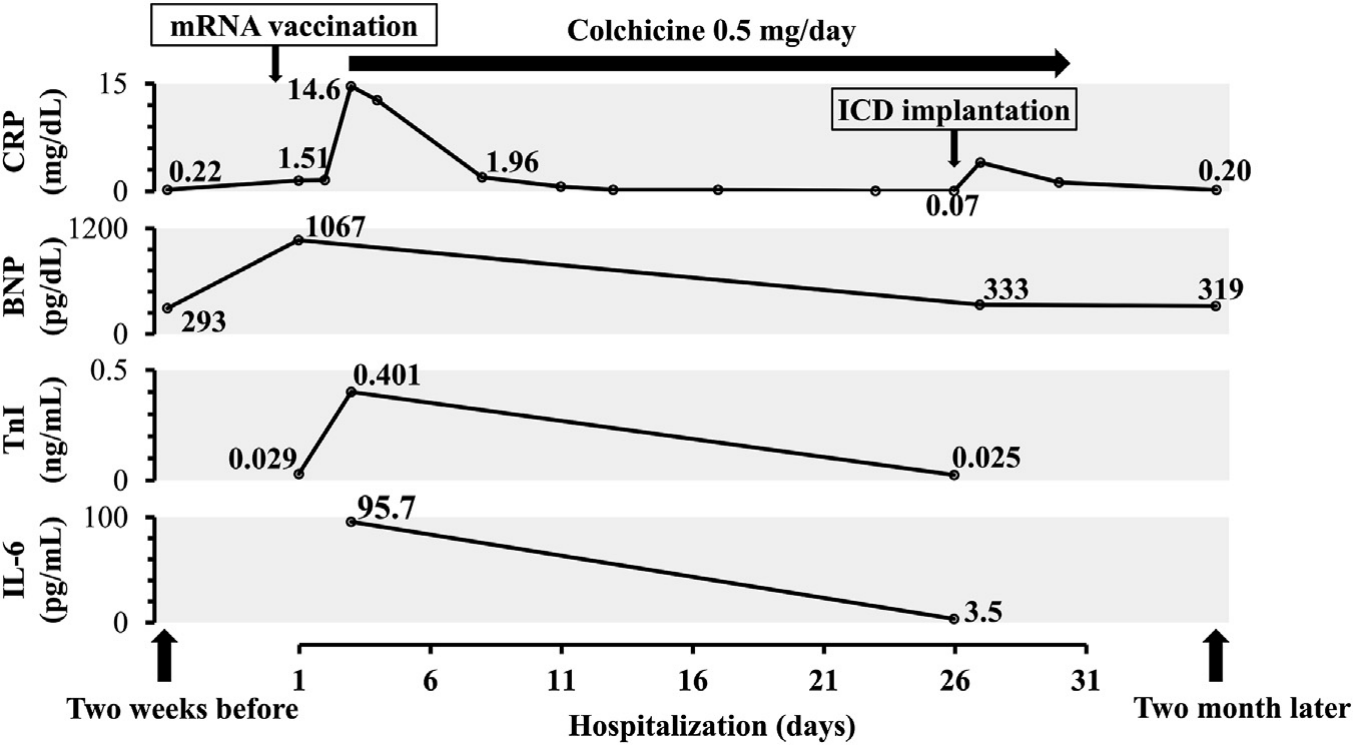
Trends in biomarker levels before, during, and after hospitalization. BNP, brain natriuretic peptide; CRP, C-reactive protein; ICD, implantable cardioverter defibrillator; IL-6, interleukin-6; TnI, high-sensitivity troponin.

He was weaned from the ventilator on day 2, but his congestive heart failure showed little improvement. On day 3, he developed chest pain and nonsustained VT with continued elevation of high-sensitivity troponin I (0.401 ng/mL) and C-reactive protein (14.63 mg/dL) levels. He also was noted to have hypercytokinemia, with an elevated IL-6 level (95.7 pg/mL; normal < 7 pg/mL). Given that new electrocardiographic changes had been seen on admission, we suspected myocarditis and administered colchicine 0.5 mg/d. An electrocardiogram obtained on day 4 showed that the right bundle branch block and ST-segment depression seen on admission had improved (Fig. 1B). Thereafter, his condition improved and the high-sensitivity troponin I, C-reactive protein, and IL-6 levels decreased (to 0.025 ng/mL, 0.07 mg/dL, and 3.5 pg/mL, respectively, on day 26; Fig. 2). On day 13, repeat CT showed no inflammatory source, and resolution of pleural effusion. On day 17, uptake of technetium-99m was decreased from the basal to the mid-inferolateral regions, in comparison with that of the fatty acid analog ^123^I-BMIPP on myocardial scintigraphy scans obtained 9 years earlier, which suggested myocardial injury (Fig. 1, C and D). Coronary angiography showed no stenosis on day 18, and a right ventricular endocardial biopsy revealed only mild fibrosis. Cardiac magnetic resonance imaging (CMR) performed on day 19 after the patient’s renal function had recovered to a level amenable to use of gadolinium contrast showed late gadolinium enhancement (LGE) in the mid layer of the anterior septum, suggestive of preexisting cardiomyopathy. However, the LGE and high signal on T2-weighted images in the inferolateral segments of the epicardial to mid layers suggested nonischemic heart disease (Fig. 1, E and F). These findings were considered compatible with myocarditis because they met the Lake Louise consensus criteria (2/3 positive).^1^ Given that the laboratory test results, chest CT, and echocardiography showed no features consistent with Fabry disease or cardiac sarcoidosis, we diagnosed this as a case of probable myocarditis due to COVID-19 vaccination. The patient was discharged home on day 31 after ICD implantation, and colchicine was discontinued. Two months later, his condition was stable, and echocardiography showed no septal thinning or new-onset left ventricular dysfunction.

## Discussion

This patient was elderly, and the time to onset of myocarditis after vaccination (1 day) was slightly shorter than the median of 2-3 days indicated in a previous report, which also indicated that myocardial infarction occurs on the day of vaccination or the following day.^2^ Myocarditis also has been reported to be more common in younger patients, and myocardial infarction more common in elderly patients.^2^ Chest pain that develops in an elderly individual within a short time after vaccination may indicate onset of myocardial infarction, which is an important differential disease. Our patient also developed congestive heart failure with VT before onset of chest pain, which is consistent with a previous report of ventricular arrhythmia occurring as the first manifestation of myocarditis.^1^

This report indicates the need to suspect myocarditis based on clinical presentation, and the importance of multimodality diagnosis using electrocardiography, echocardiography, laboratory testing, myocardial scintigraphy, and CMR.^1^ In our case, CMR showed LGE in the inferolateral segments of the epicardial to mid layers, which has been reported to be a characteristic finding in patients with mRNA vaccine-associated myocarditis.^3^ Endocardial biopsy is the gold standard for detecting myocarditis, but it is invasive and is thought to have less sensitivity in disorders resulting from epicardial and patchy diseases, such as myocarditis.^3^ On the other hand, CMR is considered to be the cornerstone for diagnosis of vaccine-associated myocarditis due to its high diagnostic performance,^3^ with a reported sensitivity of 88% and specificity of 96% in community-acquired myocarditis.^1^^,^^3^

The COVID-19 vaccine is thought to cause myocarditis via direct damage by free spike protein^4^ and induction of inflammatory cytokines (eg, IL-1β and IL-6) by the lipid nanoparticles covering the mRNA.^5^ Expression of free spike protein may increase after the initial bivalent vaccination because antibodies against the spike protein of the BA.4-5 variant are yet to be generated.

In autopsy cases, histology has shown patchy interstitial myocardial T-lymphocytic infiltration (T-cell dominant; cluster of differentiation [CD]4 > > CD8) associated with damage to myocytes.^6^ Molecular mimicry between myocyte tissue and the SARS-COV2 spike protein may also produce an anti-myocytic immune response.^6^ Therefore, T lymphocyte-mediated cell injury and heart-specific autoimmunity have been suggested as mechanisms of postvaccine myocarditis.^6^

Given that VT originating from the right ventricular outflow tract often develops via the sympathetic nervous system, β-blockers are the first-line treatment. This morphology of VT was not seen on previous Holter monitoring, suggesting that it may have been influenced by not only the left ventricular substrate as confirmed by CMR but also autonomic abnormalities caused by hypercytokinemia.

Colchicine may be an effective treatment for post-COVID-19 vaccine-associated myocarditis because it reduces susceptibility to ventricular arrhythmias by suppressing inflammation via inhibition of IL-1β, which induces IL-6.^7^ Clinicians are reluctant to use steroids to treat elderly patients with poor cardiac function, and colchicine may be a safer alternative without cardiovascular effects.

In conclusion, we propose that myocarditis after COVID-19 vaccination is difficult to diagnose and may present as a potentially fatal condition causing cardiac arrest, as in this case of poor cardiac function in which an ICD was indicated. Clinicians should be aware of the possibility of changes in a patient’s cardiovascular status after COVID-19 vaccination and the need for early diagnosis and treatment to avoid a severe adverse reaction.

**Novel Teaching Points**

• Hypercytokinemia after COVID-19 vaccination may cause myocarditis, leading to potentially fatal arrhythmia.
• Colchicine may be an effective treatment for post-COVID-19 vaccine-associated myocarditis.
• Multimodality diagnostics are important for detecting myocarditis after COVID-19 vaccination.
• Clinicians should be aware of the possibility of changes in a patient’s cardiovascular status after COVID-19 vaccination.
